# Evaluation of Risk Factors for Antibiotic Resistance in Patients with Nosocomial Infections Caused by* Pseudomonas aeruginosa*

**DOI:** 10.1155/2016/1321487

**Published:** 2016-08-30

**Authors:** Meliha Cagla Sonmezer, Gunay Ertem, Fatma Sebnem Erdinc, Esra Kaya Kilic, Necla Tulek, Ali Adiloglu, Cigdem Hatipoglu

**Affiliations:** ^1^Department of Clinic of Infectious Diseases and Clinical Microbiology, Ankara Training and Research Hospital, Ankara, Turkey; ^2^Department of Microbiology and Clinical Microbiology, Ankara Training and Research Hospital, Ankara, Turkey

## Abstract

*Background*.* Pseudomonas aeruginosa* (*P. aeruginosa*) is resistant to various antibiotics and can cause serious nosocomial infections with high morbidity and mortality. In this clinical study, we investigated the risk factors in patients who were diagnosed with* P. aeruginosa*-related nosocomial infection.* Methods*. A retrospective case control study including patients with* P. aeruginosa*-related nosocomial infection. Patients who were resistant to any of the six antibiotics (imipenem, meropenem, piperacillin-tazobactam, ciprofloxacin, amikacin, and ceftazidime) constituted the study group.* Results*. One hundred and twenty isolates were isolated. Various risk factors were detected for each antibiotic in the univariate analysis. In the multivariate analysis, previous cefazolin use was found as an independent risk factor for the development of imipenem resistance (OR = 3.33; CI 95% [1.11–10.0]; *p* = 0.03), whereas previous cerebrovascular attack (OR = 3.57; CI 95% [1.31–9.76]; *p* = 0.01) and previous meropenem use (OR = 4.13; CI 95% [1.21–14.07]; *p* = 0.02) were independent factors for the development of meropenem resistance. For the development of resistance to ciprofloxacin, hospitalization in the neurology intensive care unit (OR = 4.24; CI 95% [1.5–11.98]; *p* = 0.006) and mechanical ventilator application (OR = 11.7; CI 95% [2.24–61.45]; *p* = 0.004) were independent risk factors.* Conclusion*. The meticulous application of contact measures can decrease the rate of nosocomial infections.

## 1. Introduction

Healthcare associated infections (HAIs) (or nosocomial infections) are the worldwide public health problem causing morbidity and mortality especially in the developing countries. Furthermore, if the causative organism has developed resistance to a number of antimicrobial agents management of the issue gets harder [1]. The developing countries have taken the commendable strategies of introducing laws to control of HAIs [2].

Intensive care units (ICUs) are units where healthcare infections seen more often because of commonly critically ill patients and invasive interventions used in these units. In ICUs antimicrobial resistance rates are increasing because of various reasons such as broad spectrum and/or inappropriate antimicrobial usage and prolonged length of stay in hospital. As a result, it increases healthcare infection rates caused by multidrug resistant microorganisms. These infections prolong hospitalization, require more extensive diagnostics and treatment, and are associated with additional costs [3–5].

Device-associated healthcare associated infections (DA-HAI) are defined by the Centers for Disease Control and Prevention's (CDC's) National Healthcare Safety Network (NHSN) as infections acquired in a hospital by a patient who was admitted for a reason other than that infection [6, 7].

Worldwide view of HAIs in ICUs can be evaluated by comparing studies containing developing and developed several countries [8–10]. According to the KISS data of Germany between 2005 and 2009,* P. aeruginosa* was causative agent in 17.7% of ventilator associated pneumonia and 14.2% of urinary catheter associated urinary tract infections of all ICU infections [8]. According to the INICC data of Iran between 2011 and 2012,* P. aeruginosa* was causative agent in 19% of ventilator associated pneumonia, 5% of urinary catheter associated urinary tract infections, 2% of blood stream infections, and 7% of surgical site infections of all ICU infections [9].

The infection rates for nosocomial infections and their pathogens differ greatly between different types of ICU corresponding to the different risk structure of the patients. Different studies have been conducted to highlight the incidence and importance of hospital acquired infections in ICUs, to contribute to empirical treatment methods by determining the causes of common hospital infections and antibiotic resistance rates, to minimize the emergence of resistant microorganisms by preventing unnecessary antibiotic use, and to emphasize the need for protective measures against risk factors that favor hospital infections [5–8].

*P. aeruginosa* is an important pathogen, especially in immunocompromised patients. Besides,* P. aeruginosa* causes infections with high morbidity and mortality in intensive care units (ICUs).* P. aeruginosa* related infections are frequently life threatening and often difficult to treat due to the intrinsic resistance to many antimicrobial agents. Moreover, the resistance to antipseudomonal agents has become an increasing problem in recent years [11–14].

The present study aims to determine the risk factors for the emergence of* P*.* aeruginosa* infections those are resistant to imipenem, meropenem, piperacillin-tazobactam, amikacin, ceftazidime, or ciprofloxacin and compare the risk factors between isolates that are resistant and sensitive to each antibiotic separately. Furthermore, it aims to guide clinicians regarding treatment and infection control by revealing the relationship between antibiotic resistance and risk factors.

## 2. Material and Methods

### 2.1. Hospital Settings and Study Population

A retrospective case-control study was conducted at Ankara Training and Research Hospital in Turkey between January 2008 and July 2011. The hospital is a 670-bed referral and tertiary care hospital. The hospital contains medical and surgical ICUs. Neurology, neurosurgery, and anesthesia-reanimation ICUs with 31 total bed capacity were included in the study.

### 2.2. Study Design and Data Collection

In the hospital, nosocomial infections in ICUs have been determined by prospective, laboratory-based and patient based active surveillance since 2008. In the study, the relevant surveillance data has been evaluated to determine the risk factors for resistant* P. aeruginosa* related infections. Patients who underwent inpatient treatment in these ICUs and were diagnosed as having* P. aeruginosa *related infection 48 hours after being hospitalized were included in the study. The patients with* P. aeruginosa* resistant to selected antibiotics were defined as case groups and the patients with* P. aeruginosa* sensitive to the related antibiotic were defined as control groups.

A list of potential risk factors including the risk factors in the hospital settings was formed consistent with the relevant literature. The risk factors were as follows: gender, age, ICU type,* P. aeruginosa* as a cause of multiple sites of infections, being infected with other resistant microorganisms within 30 days before or concurrently with* P. aeruginosa* infection, existence of comorbid diseases, invasive procedures, antibiotic use, and other drugs within 30 days before the isolation of* P. aeruginosa*.

### 2.3. Microbiological Examination

All* P. aeruginosa *were isolated from various clinical specimens in the hospital microbiology laboratory by conventional biochemical methods. Recurrent isolates from the same patient were excluded from the study. The identification and antibiotic susceptibilities of the isolates were made by VITEK II automated system (Biomèrieux, France) and the results were interpreted according to standards of Clinical and Laboratory Standards Institute (CLSI) [15]. Intermediate susceptible isolates were considered to be susceptible.

### 2.4. Definitions

Nosocomial infections were defined according to the criteria proposed by the Centers for Disease Control and Prevention (CDC) [6]. The patients with nosocomial infection due to resistant (R) strains were compared with those with susceptible (S) strains for the respective antimicrobial resistances, that is, imipenem (IMP-R), meropenem (MEM-R), ceftazidime (CAZ-R), piperacillin-tazobactam (TZP-R), ciprofloxacin (CIP-R), and amikacin (AK-R). The risk factors in nosocomial infections for antimicrobial resistance to imipenem (IMP), meropenem (MEM), piperacillin-tazobactam (TZP), ceftazidime (CAZ), ciprofloxacin (CIP), and amikacin (AK) were evaluated. After the hospitalization of the patients, antibiotics that were taken 30 days before isolation of* P. aeruginosa *and used for 48 hours and longer were defined as previous antibiotic use. The elapsed time between the admission to ICU and isolation of* P. aeruginosa* was defined as the “risk period.”

### 2.5. Statistical Analysis

The SPSS 15.0 program was used for statistical analysis. The Mann–Whitney *U* test was used to compare two independent groups. The Chi-square test was used to analyze the categorical variables. In addition, the multiple logistic regression analysis was performed to determine independent risk factors that were influential on being resistant to different antibiotics. Variables included in the model were determined by using univariate statistical methods in the multivariate analysis. Variables with a significance level of *p* < 0.05 were compared with multiple logistic regression analysis. Multiple logistic regression analysis results were summarized with odds ratios, 95% confidence interval, and *p* values. In the presentation of demographic data as descriptive statistics, rates and frequency were given in qualitative variables, whereas medium (minimum-maximum) and/or mean ± standard deviation were given in quantitative variables. *p* < 0.05 was regarded as significant.

## 3. Results

### 3.1. Demographic and Clinical Features

One hundred twenty isolates that were isolated from 120 patients and met the inclusion criteria were included in the study. Thirty-four (28.3%) patients were in neurosurgery ICU, 30 (25%) patients were in Neurology ICU, and 56 (46.6%) patients were in anesthesia-reanimation ICU. During the study, 85 (70.8%) patients have been died. The hospitalization period was 4–413 days in ICU; the period until* P. aeruginosa* isolation was 3–292 days. The distribution of* P. aeruginosa* related infection types was as follows: ventilator associated pneumonia in 61 patients (50.8%), urinary system infection in 39 patients (32.5%), wound infection in 13 patients (10.8%), bloodstream infection in five patients (4.1%), and catheter infection in two patients (1.6%). Among the patients diagnosed with* P. aeruginosa* related infection, 37 (30.8) patients were transferred from another ICU of the hospital and 15 patients (12.5%) were transferred from another hospital. One hundred five (87.5%) patients used an antimicrobial within 30 days before* P. aeruginosa* isolation. The most frequently used antibiotics were carbapenem (*n* = 73, 60.8%) and meropenem (*n* = 67, 90.1%). Demographic and clinical characteristics of the study population are described in Table 1. The majority of the isolates were resistant to imipenem (45.8%), and then to meropenem and aztreonam (each with 43.3%). The isolates were mostly sensitive to colistin (100%), followed by tobramycin (80%) and amikacin (78.3%). Multiple antibiotic resistance was observed in 37 isolates (31.6%).

### 3.2. Risk Factors Associated with Antimicrobial Resistance

Various risk factors were detected for each antibiotic between sensitive and resistant* P. aeruginosa* isolates in the univariate analysis. The factors associated with antimicrobial resistance to imipenem, meropenem, and piperacillin-tazobactam are shown in Table 2 and ceftazidime, ciprofloxacin, and amikacin are shown in Table 3. For the patients with IMP-R, MEM-R, TZP-R, CAZ-R, CIP-R, and AK-R* P. aeruginosa*, the common risk factors were as follows: infection with another microorganism prior to the isolation, an ICU stay > 60 days, total parenteral nutrition usage as an invasive procedures, comorbid cerebrovascular disease, history of cerebrovascular attack, and antimicrobial use (especially meropenem) within 30 days before the isolation were performed using variables that were significantly associated with the respective antimicrobial resistance in univariate analyses (*p* < 0.05) and the identified independent risk factors are shown in Table 4. According to the analysis, independent risk factors were as follows: for imipenem resistance, previous cefazolin use; for meropenem resistance, history of cerebrovascular attack and previous meropenem use; for amikacin, stay in the ICU > 60 days. The independent risk factor associated with resistance to ciprofloxacin, piperacillin-tazobactam, and ceftazidime was history of stay in NR-ICU in multivariate logistic regression analyses.

## 4. Discussion

The present study is significant as it is a comprehensive study that investigates the risk factors in resistant* P. aeruginosa* infections in ICUs. Previous studies on carbapenem-resistant* P. aeruginosa* (CR-*Pa*) infections have shown that hospitalization in ICU is a major risk factor [16–18]. In the current study, all patients were selected from ICUs. Since the mean ICU stay was 112.7 ± 87.8 days, staying in the ICU > 60 days was evaluated as a risk factor. ICU stay > 60 days was significantly higher in patients with MEM-R*Pa* or with IMP-R*Pa* when compared to patients with MEM-S*Pa* or IMP-S*Pa*. There was no significant correlation between carbapenem resistance and type of ICU. The univariate analysis showed a significant correlation between multiple isolation of* P. aeruginosa* in the same patient (recurrent infection) as well as polymicrobial infection and imipenem or meropenem resistance. However in multivariate analyses these variables were not detected as independent risk factors. Studies on carbapenem-resistant* Pa* infections have not frequently focused on these two risk factors. The means of time at risk (until the isolation of* P. aeruginosa*) were higher in both the imipenem and meropenem-resistant group. Longer risk periods increase the ratios of infection with resistant microorganisms. The high rate of infection history with another microorganism further supports this possibility. Prolonged exposure to antibiotics in recurrent infections and the use of broad spectrum antibiotics in polymicrobial infections were considered to lead the selection of resistant microorganisms. Similar studies have shown that the mean risk period is significantly high in the imipenem-resistant group [18, 19].

When we evaluated the correlation between underlying diseases and resistance development, we did not identify any significant comorbid diseases in imipenem-resistant infections. However, the rate of cardiovascular diseases and history of SVO were significantly higher in the meropenem-resistant group. History of SVO was identified as an independent risk factor for meropenem-resistant* P. aeruginosa* infections. We believe that the incidence of meropenem-resistant strains increased, since imipenem is not preferred due to its potential convulsive effect in patients with a central nervous system (CNS) disease. However, further studies are required to support this correlation. When we examined the invasive procedures, TPN and NG catheterization were significantly higher in the imipenem-resistant group, and TPN was significantly higher in the meropenem-resistant group. However, it was not identified as an independent risk factor in the multivariate analysis. To the best of our knowledge, there have been no studies that have identified TPN and NG as independent risk factors for meropenem- or imipenem-resistant* P. aeruginosa* infections. On the other hand, some studies have linked hemodialysis, tracheostomy, arterial way, SVK, and MV to meropenem or imipenem resistance [16–18, 20, 21].

In the evaluation of previous antibiotic use, the use of cefazolin, ceftazidime, meropenem, and amikacin within 30 days before isolation was higher in the imipenem-resistant group. On the other hand, the multivariate analysis showed that only cefazolin use was an independent risk factor. Univariate analysis in the meropenem-resistant group showed higher usage rates of ceftazidime, meropenem, and amikacin; however, the multivariate analysis showed that only meropenem use was an independent risk factor. Previous studies have identified previous imipenem use as an independent risk factor for IMP-R*Pa* infection [16, 18, 21]. According to a study by Juan et al., the effects of primary resistance rates in ICU and endemic clones are low, whereas secondary resistance rates (resistance development during treatment) are high [22]. This condition highlights the importance of antibiotic use and its efficacy in resistance development. Cefazolin was preferred more frequently for surgical prophylaxis in surgical ICUs, compared to nonsurgical ICUs, and the differences in total patient numbers (87 patients in AR-ICU and NRS-ICU and 30 patients in the NR-ICU) are thought to be responsible for this outcome. Additionally, MEM is preferred more frequently than IMP in NR-ICU. Similar studies on hospital infections that are seen in general hospital populations have not identified first-generation cephalosporin use as a risk factor for carbapenem resistance [18, 19]. Furtado et al. analyzed the risk factors in pneumonia cases with IMP-R*Pa* and identified third-generation cephalosporin use as an independent risk factor [17]. Similar studies have also identified piperacillin-tazobactam use as an independent risk factor for the development of imipenem resistance, and this finding was attributed to the selection of strains with suppressed beta-lactamase production [16, 17, 19]. In the current study, ceftazidime and piperacillin-tazobactam use were significantly higher in both imipenem-resistant and meropenem-resistant groups, but these factors were not identified as independent risk factors.

The incidence of piperacillin-tazobactam resistant* P. aeruginosa* (TZP-R*Pa*) infections has increased as a result of ineffective inhibition of chromosomal beta lactamases by tazobactam; however, the incidence is lower compared to carbapenem-resistant infections [23]. In the United States, between 1998 and 2004, TZP-R*Pa* was identified as a cause of infection in ICUs (17.5%), non-ICU facilities (11.6%), and patients who receive medical care outside hospitals (6%) [24]. TZP-R*Pa* strains are responsible for 40% of all causes of hospital infections in Turkey [25]. Similar to the previous studies in Turkey, we found that 44 of 120* P. aeruginosa* strains (36.7%) strains were resistant to piperacillin-tazobactam. Different studies on resistant* P. aeruginosa* infections have shown that ICU stay is a major risk factor [26–28]. In the present study we classified our patients with respect to the type of ICU. We determined that staying in the NR-ICU as an independent risk factor for (TZP-R*Pa*) infections. The mean age of patients in NR-ICU was 67.9 ± 14.3 years and was higher compared to the other ICUs. The mean APACHE II scores (at the time of* P. aeruginosa* isolation) and the mean risk period (63.3 ± 49.1 days) were higher compared to other ICUs; however, the mean stay in NR-ICU patients (96.8 ± 61.8 days) was shorter. CVD as the primary diagnosis of majority of the patients and the higher incidence of >2 comorbid diseases support the finding that NR-ICU stay is an independent risk factor. Among the invasive interventions, TPN application had higher frequency. The use of TZP, MEM, and CIP within 30 days before isolation was significantly higher in the TZP-R*Pa* group. To date, studies have identified previous use of piperacillin-tazobactam, imipenem, aminoglycosides, vancomycin, and third-generation cephalosporins as independent risk factors [27, 28]. Similar studies of hospital infections in the same settings have identified the use of broad spectrum cephalosporins, ciprofloxacin, and fluoroquinolones within 30 days before isolation as an independent risk factor [26, 29, 30].

Studies on fluoroquinolone-resistant* P. aeruginosa* (FQ-R*Pa*) infections have shown that ICU stay is an important risk factor [26, 31, 32]. In the current study, we identified NR-ICU stay as an independent risk factor for the development of CIP-R*Pa* infections. According to the Medline database, there are no studies that investigate these risk factors in fluoroquinolone-resistant* P. aeruginosa* infections [32, 33]. In the present study, the univariate analysis showed a significant correlation between polymicrobial infections with other microorganisms at the time of* P. aeruginosa* isolation and CIP-R*Pa*. Two studies have shown that polymicrobial bacteremia is not a risk factor for antipseudomonal FQ-R*Pa* related bacteremia [26, 29]. Among the invasive interventions, mechanic ventilation was identified as an independent risk factor. Two studies have identified urinary catheterization and invasive procedures within 72 hours before bacteremia development as independent risk factors for the development of CIP-R*Pa* bacteremia [26, 29]. In another study, tracheostomy and chemotherapy have been identified as independent risk factors for the development of ciprofloxacin- and imipenem-resistant infections [32]. In this study, prior use of MEM within 30 days before isolation was identified as a risk factor. Ciprofloxacin use was higher in the resistant group, but this finding was not statistically significant. Similar studies have identified previous use of fluoroquinolones as an independent risk factor for the development of CIP-R*Pa* infections [29–31, 34]. Lee et al. found that previous levofloxacin use is an independent risk factor for* P. aeruginosa* hospital infections, but they did not determine a significant correlation between previous ciprofloxacin use [35]. On the other hand, two different studies identified a significant correlation between previous carbapenem and fluoroquinolone use and resistance development [26, 36]. In the current study, ciprofloxacin use was higher in the resistant group, but this finding was not statistically significant.

According to the Medline database, there are only a limited number of epidemiological studies on aminoglycoside resistance of* P. aeruginosa*. In the current study, the univariate analysis of risk factors that favored AK-R*Pa* infections showed that ICU stay > 60 days, thoracotomy tube, and prior meropenem use were significantly higher in the resistant group. Among these risk factors, we identified the thoracotomy tube as an independent risk factor. To the best of our knowledge, there are no previous studies that investigated the application of a thoracotomy tube as a potential risk factor. According to a study on risk factors in AK-R*Pa* related bacteremia, the use of fluoroquinolones within 90 days before isolation, urinary catheterization, and percutaneous catheterization are independent risk factors [29]. Another study indicated that risk factors that lead to gentamicin resistance in* P. aeruginosa*, previous gentamicin use, and multiple antibiotic use are independent risk factors [37]. Another study indicated that previous use of meropenem and amikacin is independent risk factors for AK-R*Pa* [37]. Previous use of first and third-generation cephalosporins and piperacillin is independent risk factors for CAZ-R*Pa *infections in a similar study [35]. Fortaleza et al. found that previous amikacin use is an independent risk factor for the development of CAZ-R*Pa *infections [18]. In the current study NR-ICU stay was the only independent risk factor for the development of CAZ-R*Pa*. The underlying causes of NR-ICU stay being an independent risk factor were mentioned in the section of TZP-R*Pa*. The same causes are also independent risk factors for the development of CAZ-R*Pa*. In addition, we found a significant correlation between polymicrobial infections before isolation of* P. aeruginosa *and the development of CAZ-R*Pa*. In a similar study, polymicrobial infection is not a risk factor for the development of CAZ-R*Pa*; however, it is an independent risk factor for mortality [29]. Comorbid cardiovascular diseases and decubitus ulcer were significant for the development of CAZ-R*Pa* infections. Cardiovascular diseases (92.1%) were the most common comorbid disease in the resistant group. Two studies on risk factors that affect the development of resistance to antipseudomonal antibiotics in* P. aeruginosa* related bacteremia have identified solid tumors as the most common underlying disease; however, this was not identified as a risk factor [26, 29]. Some of the previous studies have demonstrated that urinary catheterization, percutaneous catheterization, and invasive intervention within 72 hours before isolation are independent risk factors [34] on the contrary of this study. We determined that invasive procedures and medical interventions were not risk factors for the development of ceftazidime resistance. Some of the previous studies have demonstrated that urinary catheterization, percutaneous catheterization, and invasive intervention within 72 hours before isolation are independent risk factors [29]. The frequency of surgical operations in the ceftazidime-sensitive group was significantly higher compared to the ceftazidime-resistant group.

The effective treatment of infections caused by* P. aeruginosa* includes prevention when possible and source control measures as necessary and prompt administration of appropriate antimicrobial agents. If antimicrobial susceptibilities are known, deescalation should be pursued in patients especially with an appropriate clinical response. Hand hygiene and barrier precautions are important to keep the spread of infection in ICUs. Therefore surveillance is important in providing useful information for physicians in choosing empirical antibiotics [38–40].

## 5. Conclusion

To date, most of the studies including the present study have indicated the fact that long-term hospitalization of patients with poor overall condition with multiple invasive procedures and intense antibiotic pressure (especially carbapenem-class antibiotics) have led to resistant* P. aeruginosa* infections.

These results should be taken into consideration to comprehend the importance of limiting antibiotic use in order to prevent resistance to antibiotics that can be used for the treatment of life threatening infections.

## Figures and Tables

**Table 1:** Demographic and clinical characteristics of patients with *P. aeruginosa *related nosocomial infection.

Characteristics	Number of patients (%) (*n*: 120)
Age; years (mean ± SD)	58.4 ± 19.2
>60 years old	70–58.3
Male/female	62–50.8/58–49.2
Intensive care unit (ICU)	
^a^NR-ICU	34–28.3
^b^AR-ICU	56–46.6
^c^NRS-ICU	34–28.3
Stay at ICU (mean ± SD)	112.7 ± 87.8
Intensive care unit stay > 60 days	82–68.3
Polymicrobial infection	90–75
Multiple isolation of *P. aeruginosa*	50–41.7
Time at risk (mean ± SD)	55.4 ± 52.4
APACHE II score (mean ± SD) (at time of isolation)	23.6 ± 4.14
Invasive procedures and comorbid disease	
Mechanic ventilation	96–80
Enteral nutrition	105–87.5
Total parenteral nutrition	87–72.5
Thoracotomy tube	17–14.2
Urinary catheterization	119–99.2
Central venous catheterization	108–90
History of cerebrovascular disease	78–65
History of cardiovascular disease	98–81.7
History of surgical operation	40–33.3
History of chronic renal disease	32–26.7
History of malignancy	69–57.5
Prior antibiotic use	
Carbapenems	73–60.8
Meropenem	67–90.1
Piperacillin-tazobactam	50–41.7
Amikacin	44–36.7
Teicoplanin	38–31.7
Cefazolin	29–24.2
Ceftriaxone	22–18.3
Ciprofloxacin	10–8.3
Ceftazidime	9–7.5

^a^NR-ICU: neurology intensive care unit, ^b^AR-ICU: anesthesia-reanimation intensive care unit, and ^c^NRS-ICU: neurosurgery intensive care unit.

**Table 2:** Univariate analysis of risk factors for antimicrobial resistance in nosocomial infections due to *P. aeruginosa *(IMP, MEM, and TZP).

Risk factors	^1^IMP-^*∗*^R (*n* = 56) (*n*-%)	IMP-^*∗∗*^S (*n* = 64) (*n*-%)	*p*	^2^MEM-R (*n* = 52) (*n*-%)	MEM-S (*n* = 68) (*n*-%)	*p*	^3^TZP-R (*n* = 44) (*n*-%)	TZP-S (*n* = 76) (*n*-%)	*p*
>60 years old	31–55.4	39–60.9	0.536	27–51.9	43–63.2	0.213	26–59.1	44–57.9	0.898
Sex (male)	28–50	33–51.6	0.864	24–46.2	35–51.5	0.564	20–45.5	41–53.9	0.370
Intensive care unit type									
^a^NR-ICU	14–25	16–25	0.782	16–30.8	14–20.6	0.206	20–45.5	10–13.2	**<0.001**
^b^AR-ICU	25–44.6	32–50		20–38.5	37–54.4		17–38.6	40–52.6	
^c^NRS-ICU	17–30.4	16–25		16–30.8	17–25		7–15.9	26–34.2	
Intensive care unit stay > 60 days	47–83.9	35–54.7	**0.001**	46–88.5	36–52.9	**<0.001**	34–77.3	48–63.2	0.109
Polymicrobial infection	49–87.5	41–64.1	**0.003**	46–88.5	44–64.7	**0.003**	38–86.4	52–68.4	**0.029**
Multiple isolation of *P. aeruginosa*	34–60.7	16–25	**<0.001**	32–61.5	18–26.5	**<0.001**	19–43.2	31–40.8	0.798
Time at risk > 30 days	43–76.8	36–56.2	**0.018**	40–76.9	39–57.4	**0.024**	32–72.7	47–61.8	0.226
APACHE II score (mean ± SD) (at time of isolation)	24.1 ± 5.8	23.1 ± 6.6	0.435	24 ± 5.6	22.2 ± 4.6	0.739	24.6 ± 6.3	23 ± 6.1	0.164
Mechanic ventilation	48–85.7	48–75	0.143	44–84.6	42–72.5	0.269	38–86.4	58–76.3	0.185
Enteral nutrition	53–94.6	52–81.3	**0.027**	48–92.3	57–83.8	0.164	40–90.9	65–85.5	0.390
Total parenteral nutrition	47–83.9	40–62.5	**0.009**	43–82.7	44–64.7	**0.029**	38–86.4	49–64.5	**0.010**
Thoracotomy tube	9–16.1	8–12.5	0.576	7–13.5	10–14.7	0.846	7–15.9	10–13.2	0.677
History of cerebrovascular disease	40–71.4	38–59.4	0.167	42–80.8	36–52.9	**0.002**	30–68.2	48–63.2	0.578
History of cardiovascular disease	49–87.5	49–76.6	0.122	47–90.4	51–75	**0.031**	39–88.6	59–77.6	0.133
History of surgical operation	21–37.5	19–29.7	0.365	18–34.6	22–32.4	0.794	9–20.5	31–40.8	**0.023**
Prior receipt of cefazolin	19–33.9	10–15.6	**0.019**	14–26.9	15–22.1	0.537	7–15.9	22–28.9	0.108
Prior receipt of ceftazidime	8–14.3	1–1.6	**0.008**	7–13.5	2–2.9	**0.030**	2–4.5	7–9.2	0.350
Prior receipt of meropenem	42–75	25–39.1	**<0.001**	42–80.8	25–36.8	**<0.001**	31–70.5	36–47.4	**0.014**
Prior receipt of amikacin	28–50	16–25	**0.005**	26–50	18–26.5	**0.008**	18–40.9	26–34.2	0.463
Prior receipt of piperacillin-tazobactam	26–46.4	24–37.5	0.322	25–48.1	25–36.8	0.213	24–54.5	26–34.2	**0.029**
Prior receipt of ciprofloxacin	4–7.1	6–9.4	0.659	7–13.5	3–4.4	0.076	7–15.9	3–3.9	**0.022**
Prior receipt of teicoplanin	25–44.6	13–20.3	**0.004**	20–38.5	18–26.5	0.162	16–36.4	18–23.7	0.137

^a^NR-ICU: neurology intensive care unit, ^b^AR-ICU: anesthesia-reanimation intensive care unit, and ^c^NRS-ICU: neurosurgery intensive care unit.

^1^IMP: imipenem, ^2^MEM: meropenem, and ^3^TZP: piperacillin-tazobactam.

^*∗*^R: resistant and ^*∗∗*^S: sensitive.

**Table 3:** Univariate analysis of risk factors for antimicrobial resistance in nosocomial infections due to *P. aeruginosa *(CIP, AK, and CAZ).

Risk factors	^1^CIP-^*∗*^R (*n* = 40) (*n*-%)	CIP-^*∗∗*^S (*n* = 80) (*n*-%)	*p*	^2^AK-R (*n* = 26) (*n*-% )	AK-S (*n* = 94) (*n*-% )	*p*	^3^CAZ-R (*n* = 38) (*n*-% )	CAZ-S (*n* = 82) (*n*-% )	*p*
>60 years old	22–55	48–60	0.600	14–53.8	56–59.6	0.600	25–65.8	45–54.9	0.259
Sex (male)	21–52.5	40–50	0.796	13–50	48–51.1	0.923	17–44.7	44–53.7	0.363
Intensive care unit type									
NR-ICU^a^	15–37.5	15–18.8	**0.025**	9–34.6	21–22.3	0.356	19–50	11–13.4	**<0.001**
AR-ICU^b^	18–45	39–48.8		12–46.2	45–47.9		14–36.8	43–52.4	
NRS-ICU^c^	7–17.5	26–32.5		5–19.2	28–29.8		5–13.2	28–34.1	
Intensive care unit stay > 60 days	31–77.5	51–63.6	0.127	24–92.3	58–61.7	**0.003**	28–73.7	54–65.9	0.391
Polymicrobial infection	35–87.5	55–68.8	**0.025**	23–88.5	67–71.3	0.073	33–86.8	57–69.5	**0.041**
Multiple isolation of *P. aeruginosa*	19–47.5	31–38.8	0.359	12–46.2	38–40.4	0.600	18–47.4	32–39	0.388
Time at risk > 30 days	20–76.9	58–61.7	0.150	21–80.8	58–61.7	0.070	26–68.4	53–64.6	0.684
APACHE II score (mean ± SD) (at time of isolation)	24.9 ± 6.9	22.9 ± 5.8	0.104	24.3 ± 6.9	23.4 ± 6.1	0.523	25.3 ± 6.4	22.8 ± 6.0	**0.039**
Mechanic ventilation	38–95	58–72.5	**0.004**	23–88.5	73–77.3	0.223	30–78.9	66–80.5	0.844
Enteral nutrition	38–95	67–83.8	0.079	25–96.2	80–85.1	0.132	34–89.5	71–86.6	0.656
Total parenteral nutrition	32–80	55–68.8	0.193	22–84.6	65–6.1	0.118	31–81.6	56–86.3	0.129
Thoracotomy tube	7–17.5	10–12.5	0.459	7–26.9	10–10.6	**0.035**	4–10.5	13–15.9	0.436
History of cerebrovascular disease	28–70	50–62.5	0.417	17–65.4	61–64.9	0.963	28–73.7	50–61	0.175
History of cardiovascular disease	35–87.5	63–78.8	0.243	22–84.6	76–80.9	0.661	35–92.1	63–76.8	**0.044**
History of surgical operation	10–25	30–37.5	0.171	4–15.4	36–38.3	**0.028**	5–13.2	35–42.7	**0.001**
Prior receipt of cefazolin	8–20	21–26.3	0.451	5–19.2	24–25.5	0.507	5–13.2	24–29.3	0.055
Prior receipt of ceftazidime	1–2.5	8–10	0.141	1–3.8	8–8.5	0.424	2–5.3	7–8.5	0.527
Prior receipt of meropenem	28–70	39–48.8	**0.027**	20–76.9	47–50	**0.014**	26–68.4	41–50	0.059
Prior receipt of amikacin	16–40	28–35	0.592	12–46.2	32–34	0.257	16–42.1	28–34.1	0.400
Prior receipt of piperacillin-tazobactam	20–50	30–37.5	0.190	13–50	37–39.4	0.330	20–52.6	30–36.6	0.091
Prior receipt of ciprofloxacin	6–15	4–5	0.062	3–11.5	7–7.4	0.504	6–15.8	4–4.9	**0.044**
Prior receipt of teicoplanin	14–35	24–30	0.579	9–34.6	29–30.9	0.715	12–31.6	26–31.7	0.989

^a^NR-ICU: neurology intensive care unit, ^b^AR-ICU: anesthesia-reanimation intensive care unit, and ^c^NRS-ICU: neurosurgery intensive care unit.

^1^CIP: ciprofloxacin, ^2^AK: amikacin, and ^3^CAZ: ceftazidime.

^*∗*^R: resistant and ^*∗∗*^S: sensitive.

**Table 4:** Independent risk factors associated with the perspective antimicrobial resistances in resistant to antipseudomonal antibiotics related to nosocomial *P. aeruginosa *infections.

Variables	Adjusted OR (95% CI)	*p*
*Resistance to imipenem *		
Prior receipt of cefazolin	3.33 (1.11–10.0)	0.03
*Resistance to meropenem *		
History of cerebrovascular disease	3.57 (1.31–9.76)	0.01
Prior receipt of meropenem	4.13 (1.21–14.07)	0.02
*Resistance to piperacillin-tazobactam *		
Stay at neurology intensive care unit	4.47 (1.69–11.84)	0.003
*Resistance to ciprofloxacin *		
Stay at neurology intensive care unit	4.24 (1.5–11.98)	0.006
Mechanic ventilation	11.7 (2.24–61.45)	0.004
*Resistance to amikacin *		
Intensive care unit stay > 60 days	7.27 (1.60–33.02)	0.01
Thoracotomy tube	3.41 (1.03–11.24)	0.04
*Resistance to ceftazidime *		
Stay at neurology intensive care unit	5.07 (1.92–13.34)	0.001
